# Challenges and opportunities for using population health data to investigate cancer survivors’ quality of life in Australia

**DOI:** 10.1007/s11136-022-03112-3

**Published:** 2022-03-04

**Authors:** Imogen Ramsey, Nadia Corsini, Amanda Hutchinson, Julie Marker, Marion Eckert

**Affiliations:** 1 Rosemary Bryant AO Research Centre, UniSA Clinical & Health Sciences, University of South Australia, City East Campus, GPO Box 2471, Adelaide, SA Australia; 2 UniSA Justice & Society, University of South Australia, Adelaide, SA Australia; 3 Cancer Voices South Australia, Adelaide, SA Australia

**Keywords:** Quality of life, Population health, Cancer, Surveillance

## Abstract

There is a recognised need for reported national data that inform health policy, health professions, and consumers about the wellbeing of Australians with cancer and other chronic conditions. International initiatives have demonstrated the viability and benefits of utilising population-based cancer registries to monitor the prevalence and trajectory of health-related quality of life (HRQOL) outcomes among people with cancer. Establishing a similar level of monitoring in Australia would require timely access to health data collected by publicly funded, population-based cancer registries, and the capacity to link this information across jurisdictions. Combining information from different sources via data linkage is an efficient and cost-effective way to maximise how data are used to inform population health and policy development. However, linking health datasets has historically been highly restricted, resource-intensive, and costly in Australia due to complex and outdated legislative requirements, duplicative approval processes, and differing policy frameworks in each state and territory. This has resulted in significant research waste due to underutilisation of existing data, duplication of research efforts and resources, and data not being translated into decision-making. Recognising these challenges, from 2015 to 2017 the Productivity Commission investigated options for improving data availability and use in Australia, considering factors such as privacy, security, and intellectual property. The inquiry report recommended significant reforms for Australian legislation, including the creation of a data sharing and release structure to improve access to data for research and policy development purposes. This paper discusses (1) opportunities in HRQOL research enabled by data linkage, (2) barriers to data access and use in Australia and the implications for waste in HRQOL research, and (3) proposed legislative reforms for improving data availability and use in Australia.

## Background

Globally, population-based health data are being utilised to advance our understanding of trends in population health and generate timely insights for clinical practice and policy. However, in Australia, the use of population-based surveillance datasets for research has been hampered by the complexity and duplication of state and national legislation and regulations regarding data access and linkage. As a result, we have not optimised the use or social benefits of health data for improving the health-related quality of life (HRQOL) of people affected by cancer or other chronic diseases.

Waste has been defined as “non-value” and “all efforts that do not add value” [1]. In its 2014 series “Increasing value, reducing waste”, The Lancet examined sources of avoidable waste in biomedical research, which were first highlighted by Chalmers and Glasziou in 2009 [2]. These sources of waste included studies that overlook important research questions [3]; that are poorly designed or conducted [4]; that are inefficiently regulated and managed [5]; that produce inaccessible information [6]; and that are not appropriately reported, disseminated, or translated into decision-making [7]. This paper focuses on inefficient research regulation and management practices as sources of various types of research waste. In addition to consuming valuable research resources, these processes prevent data from being accessed in a timely manner or at all, and therefore from being used to create value by advancing research or informing decision-making. Transparency and access to data was one of six quality dimensions agreed upon by international stakeholders for inclusion in the INcreasing QUality In patient-oriented academic clinical REsearch (INQUIRE) framework, which was developed to assist researchers in navigating the challenges of reducing waste and increasing value [8]. Ensuring that regulation and management are proportionate to risks is one of five priorities in The Lancet’s REduce Waste And Reward Diligence (REWARD) campaign, which invites funders, regulators, organisations, publishers, researchers and other stakeholders to commit to reducing waste and maximising efficiency [9].

There is a recognised need for population-based data on the long-term HRQOL experienced by people with cancer [10]. This growing population is at risk of chronic health problems; comorbidity; adverse physical, psychological, and social outcomes; secondary cancers; and long-term and late effects of treatment, all of which can significantly impact on HRQOL [11, 12]. HRQOL is inversely associated with behavioural and lifestyle risk factors and frequently predicts morbidity, mortality, and healthcare utilisation more accurately than objective health measures [13]. Therefore, its surveillance at a population level can support disease burden prediction, resource allocation, and identification of public health issues [13]. Gaining an understanding of the prevalence and trajectory of HRQOL outcomes at a population level, and how they differ based on sociodemographic and clinical factors, can inform clinical practice, health policy, the design of appropriate health interventions and services, and the allocation of health resources to areas of highest priority [14].

In Australia, population-level data on cancer incidence, mortality, and survival are collected by publicly funded, population-based cancer registries, allowing for national reporting of these outcomes over time [10]. However, these indicators represent only a fraction of the impact of cancer on population health. We need an understanding of not just length of survival, but the long-term HRQOL experienced by people with cancer, to design health services and policy that meet their needs. Collecting HRQOL data using patient-reported outcomes (PROs; self-reports about a patient’s health provided directly by the patient) at a population level presents a challenge, as this is typically beyond the remit or mandate of cancer registries [15]. In addition, legislative barriers to timely data linkage have inhibited the sharing of clinical registry data that could inform population-based HRQOL research in Australia, stifling innovation in this area. This paper discusses (1) opportunities in HRQOL research enabled by data linkage, using an international example; (2) barriers to data access and use in Australia and the implications for waste in HRQOL research; and (3) proposed legislative reforms.

## Opportunities in HRQOL research enabled by data linkage

Data linkage is the process of uniting data pertaining to an individual, group, location, or event, from multiple independent data sources, in a way that maintains privacy and confidentiality [16]. Combining data from different sources is an efficient and cost-effective way to maximise the use of existing data for informing population health and policy development, thereby reducing research burden and duplication of effort [16]. Additionally, data linkage can make detailed longitudinal patient data available and provide the infrastructure to investigate research questions requiring the use of multiple datasets [16, 17]. In the context of HRQOL research in cancer, linkage with datasets held by various levels of government can be used to examine associations of HRQOL with cancer incidence and mortality data, clinical data, and sociodemographic characteristics.

Internationally, a small number of initiatives have demonstrated the viability and benefits of collecting PROs from people with cancer utilising population-based cancer registries and linking this information with clinical and sociodemographic data, to understand the impact of cancer on HRQOL at a national scale [18]. In a systematic review, we identified seven international registries that had been established for ongoing surveillance of HRQOL among defined populations of cancer survivors. The review findings highlighted the viability of using population-based cancer registries to collect PRO data from cancer survivors. The data generated by these systems have been used for a variety of purposes, including informing disease management, clinical decision-making, health care planning, quality improvement, and understanding the impacts of cancer on population health [18].

A unique and well-established example of HRQOL surveillance is the Patient-Reported Outcomes Following Initial treatment and Long-term Evaluation of Survivorship (PROFILES) registry [19], which has collected comprehensive, longitudinal PRO data about physical, psychological, and social aspects of HRQOL from cancer survivors in the Netherlands since 2009 [18]. People diagnosed with cancer are invited by their treating physician to complete a voluntary questionnaire annually, for up to five years from primary treatment completion, allowing outcomes to be monitored over time [19]. Through data linkage with the Netherlands Cancer Registry, which records clinical information about all cancers diagnosed in the Netherlands, PROs are linked with, and interpreted in relation to, participants’ clinical and sociodemographic characteristics [19].The comprehensive datasets generated by the PROFILES registry have enabled trends in HRQOL to be monitored longitudinally at a population level, and according to factors such as tumour type and stage, age, treatment received, and comorbidity. Additionally, the availability of corresponding PRO data from a normative non-cancer population allows comparison of HRQOL between population-based cohorts of cancer survivors and demographically matched controls [19].

Research studies analysing data from the PROFILES registry have generated invaluable insight into the course of HRQOL experienced by cancer survivors in the Netherlands, documented in over 100 research studies that explored and compared outcomes among different subgroups, and for different interventions. For example, these studies demonstrated that cancer survivors reported persistently lower HRQOL and functioning [20–22], and higher prevalence of fatigue [23–26], anxiety, and depressive symptoms [27, 28] than individuals without cancer. Moreover, they have identified associations between outcomes including fatigue, depressive symptoms, and poor HRQOL with all-cause mortality [29–31]. Besides linkage with clinical cancer registry data, PROFILES data have been linked with clinical trials data to examine possible trial effects on the long-term HRQOL of cancer survivor cohorts [15], and with pharmaceutical data to determine the impacts of medications on long-term HRQOL [15]. In addition to enhancing understanding of the long-term impacts of cancer on HRQOL and opening new fields of research [15], HRQOL research studies utilising linked data have informed practice and policy for cancer survivors in the Netherlands [18]. For example, findings from the PROFILES registry have facilitated change in diverse areas including chemotherapy regimens, models of supportive care, information provision, and travel insurance [18].

## Barriers to data access and use, and implications for waste in HRQOL research

Compared with practices for data sharing in the Netherlands, linkage and integration of datasets is inadequate in Australia, where barriers to accessing and linking data sources for research purposes have been well documented [16, 32–35]. For a combination of legal, technical, and institutional reasons, Australia has historically used health information poorly compared with other developed countries [36]. Factors that restrict Australia’s sharing of data include complex legislative requirements, overly risk-averse approval processes, the lack of a whole of government approach, and differing policy frameworks in each jurisdiction [37]. The combination of these factors has resulted in research waste due to lengthy and complex processes that consume valuable research resources, duplication of research efforts, and research data not being used to address important research questions or translated into decision-making to benefit the public.

To improve data linkage and sharing within Australia, the Population Health Research Network (PHRN) was established in 2009 [34]. This network comprises national data linkage units in all Australian jurisdictions, which have the capacity to provide linked data on a national scale [34, 38]. This investment in Australian data linkage infrastructure has improved access to linked data [39] and seen an increase in the number of research publications involving the use of linked health and human services data, from 72 publications in 2009–2010 to 199 in 2016–2017 [34]. Studies using PHRN linked data have influenced health policy and service provision in the areas of cancer, diabetes, heart and cardiovascular disease, and paediatric health [40]. The results from one study that used linked data, for example, prompted changes to clinical guidelines and education about cancer risk and dosage and exposure practices for computed tomography scans [41]. The contribution of PHRN-related data to reductions in cancer burden (i.e. projected disability-adjusted life years resulting from cancer) in Australia has been estimated to exceed 0.5% by 2034, associated with a net economic benefit of $7.1 billion [42]. Despite growth in publications from research using linked data over the past 8 years, major barriers to the timely and effective use of data linkage in Australia remain [34, 38]. The jurisdictional data linkage facilities were established along with governance protocols to protect individual privacy and data confidentiality to the highest possible ethical standard [32]. However, approval processes that previously existed to protect privacy and confidentiality remained in place [32]. Researchers are therefore required to obtain multiple approvals including from data linkage centres, data custodians, and ethics committees [32]. Due to the complexity, duplication, and lack of cohesion of the procedures required to undertake data linkage, it remains a time consuming, costly, and resource-intensive process, particularly linkage of cross-jurisdictional datasets [32, 35]. For a national study, obtaining required approvals can require liaison with numerous data custodians, up to seven data linkage centres, and more than 10 ethics committees [32], with expected timelines exceeding three years [16].

As stated in Cancer Australia’s National Cancer Data Strategy [10] “while due regard for ethical standards and privacy protocols is very important, the need for multiple clearances and multiple approval processes is a major source of inefficiency and often, a barrier to progress” [10]. Indeed, while the increasing number of data sources used for population health research necessitates due consideration of consumers’ privacy and confidentiality [13], the time and resources expended in achieving data linkage undermine the potential value of research for population health [32]. The inefficiencies associated with data linkage in Australia are exacerbated by the legal requirement that datasets resulting from linked Commonwealth data (e.g. from the Pharmaceutical Benefits Program or Medicare Benefits Scene) be destroyed once a specific project is completed [37]. The waste inherently associated with the regular destruction and recreation of datasets that are linked at a high cost is significant.

Despite a decade of investment in infrastructure and funding, Australian health data are still an underutilised resource due to poor data sharing integration practices [33]. A consequence of the barriers cited is that they create disincentives to invest in systems similar to the PROFILES registry that would allow HRQOL to be assessed at a population level, to answer questions that underpin prudent cancer control strategies. These opportunity costs represent “unnoticed and unquantifiable waste, such that important research is identified but never addressed” [5]. Instead of investing in HRQOL data infrastructure to support multiple studies, there is currently a reliance on collecting data de novo for discrete studies, resulting in potential duplication of resources across the research pipeline and studies being underpowered. Ultimately, these sources of waste limit our ability to answer questions about population health and translate research data into meaningful policy action [44]. In 2008, Cancer Australia called for the gap in HRQOL data to be addressed as part of its National Cancer Data Strategy [10] but, because of these barriers, we are no closer to bridging this gap in 2021.

Waste caused by inaccessible data and inefficiencies in regulatory processes are not unique to Australia, as The Lancet’s series highlighted [5]. Internationally, governments and organisations are seeking to streamline processes and enable greater sharing of data across public and private sectors, to encourage innovation and improvement in services across various industries, including health [43]. In the United Kingdom (UK), 21 academic and health institutions formed a dedicated research institute, the Farr Institute (now Health Data Research UK), to facilitate the safe use of electronic health records and administrative data for research purposes. The Farr Institute facilitated the use of population-based datasets in 593 published papers between 2013 and 2018, a time of profound societal and policy change within the UK in relation to health data science [45]. In 2018, European Union (EU) Member states launched Information for Action (InfAct), a joint initiative to improve the availability of health data and strengthen health information activities across Europe [46]. An InfAct study published in 2020 reported that data linkage is performed widely across EU countries for health status monitoring, policy development, and scientific research [47]. However, the authors found that more flexible governance frameworks, specific mandates to ensure data availability, and consistent interpretation of the EU General Data Protection Regulations (GDPR) are required to support and enhance data linkage capabilities [47].

## Legislative reforms: a solution?

Realising the potential for national data linkage could enable valuable insights into HRQOL outcomes and their associations with medical treatments, health services, and demographic characteristics; information that is currently not available in Australia. In response to the reported challenges of data linkage, Australian researchers have called for more streamlined application and approval processes, less duplication, greater collaboration between jurisdictions, and increased infrastructure and funding support [16, 32–34]. Because consumers have the most to gain from reductions in research waste and inefficiency, they too should be involved in decisions about the need for, and extent of, the effects of regulation and management on research [5]. Support for enabling better use of Australia’s health data in research appears to be evident among consumers, who are willing to share data in exchange for outputs that are meaningful [48]. In a recent survey, 83% of participants indicated they were willing to share their de-identified health data to advance medical research [49], consistent with international evidence that most patients consent to the use of secondary data for record linkage [50]. Australian consumers have reported that they are more willing to share data when it is being used for individual or public good, and when they understand how it is being used [48]. However, consumers share differing views and understand the concept of data linkage to differing extents, highlighting the need for transparent and two-way communication about the processes, benefits, and risks involved [48]. A whole of government approach to using health data, that is co-designed with consumers, has been identified as key to increasing the consistency, quality, and value of data linkage and use in Australia [48].

Recognising the need to improve opportunities for data access and use in Australia while considering factors such as privacy, security, and intellectual property, from 2015 to 2017 the Productivity Commission conducted a broad investigation into the benefits and costs of options for improving data availability and use [37]. The inquiry report recommended significant changes for Australia’s open government agenda and the rights of consumers to data, to catch up with achievements in competing economies such as the UK, the US, New Zealand, Canada, France, and The Netherlands [37]. A key component of the recommended reforms was the creation of a data sharing and release structure, to provide better and more timely access to data for research and policy development purposes [37]. The proposed new legislation for data sharing and release has been developed by the Office of the National Data Commissioner, through extensive stakeholder consultation [51]. The proposed laws will overwrite some 500 data secrecy and confidentiality provisions in 175 pieces of existing legislation [52]. However, data will only be released if it is related to improving policy, program evaluation, service delivery and research and development [52].

How the proposed laws will translate into policy to inform health research, and whether they will help to reduce waste and increase value and opportunities for innovation in HRQOL research remains to be seen. As the Productivity Commission’s 2017 inquiry report found, a risk-averse culture may pose a greater threat to data sharing and access than legislation itself [37]. In the past, uncertainty among data collectors and custodians about how to interpret legislation such as the Privacy Act has led to overly cautious approaches to data management and complex and inefficient approval processes designed to meet legislative requirements [37]. Avoiding the same pitfalls in the reform of Australia’s data infrastructure will therefore require not only a simplified legislative framework, but a substantial shift away from an entrenched culture of risk aversion [37].

In 2018, Ford and colleagues reported similar challenges in the UK, following six years of sustained investment in improving use of health data [53]. Obstacles to data access were cited as one of the key barriers to realising an inclusive, transparent, and innovative vision for health data science [53]. Specifically, the authors reported that data access was hampered by inconsistent use of national standards, despite the introduction of legislation designed to facilitate data science, such as the GDPR and the UK Digital Economy Act 2017 [53]. Similar to the Productivity Commission’s Australian findings, Ford and colleagues linked these barriers to a broader culture of risk aversion in the UK, perpetuated by inconsistencies in how legislation is interpreted and applied [53].

In February 2021, the UK government announced a review into the efficient and safe use of health data for research to complement a proposed data strategy for health and social care. The draft strategy (*Data saves lives: reshaping health and social care with data (draft)*), released in June 2021, outlines the Secretary of State’s vision for using data to improve population health [54]. Proposed legislative changes are among an extensive list of commitments made in the report, with others including improving information governance, developing guidance and frameworks for data sharing and transparency, enabling members of the public to access their own health data, providing education and training for staff, reducing data collection burden, building analytical and data science capability, working with stakeholders and the public to implement legislative reform, modernising data architecture, and supporting innovation [54]. In outlining these commitments, the draft strategy proposes to support the creation of a strong data infrastructure and the implementation of not only legislative, but behavioural, cultural, organisational, and policy change.

## Conclusions

Inefficient regulation and management of health data creates the types of waste that Al-Shahi Salman and colleagues argue arise “from questions being overlooked or unnecessarily addressed, research being underpowered or done too slowly, and research being too costly” [5]. Consistent with global trends, there is a national imperative to achieve efficient and timely health data linkage, to optimise how data are used to inform and improve the provision of health services in Australia. The projected increase in the number of people diagnosed with, and surviving, cancer will create greater future demand across health systems. In the context of this challenge, understanding the long-term impacts of cancer on HRQOL will become increasingly important to direct resources to where they can make the most difference. Australia’s proposed legislative reforms are designed to maximise the value of public sector data for service delivery and research. It is hoped that the implementation of these reforms will reduce the fragmentation and duplication of research activities, enable more timely sharing of data for research, and expose existing data to a wider range of innovative ideas and approaches, to increase value and reduce waste. However, as the UK’s new data strategy suggests, successful implementation will require the complex task of fostering a culture of data sharing and transparency. Data are wasted if they are collected and not used to influence decision-making. Increasing the availability and use of data will ultimately enable more research to add to international literature on population health, and inform evidence-based decision-making for clinical practice, service design, and health policy. Progress across these areas is critical for improving health systems and HRQOL outcomes for the global population affected by cancer and other chronic diseases.

## Data Availability

Not applicable.
